# Maternal early mid-pregnancy adiponectin in relation to infant birth weight and the likelihood of being born large-for-gestational-age

**DOI:** 10.1038/s41598-023-48027-2

**Published:** 2023-11-27

**Authors:** Emelie Lindberger, Anders Larsson, Theodora Kunovac Kallak, Inger Sundström Poromaa, Anna-Karin Wikström, Anna Österroos, Fredrik Ahlsson

**Affiliations:** 1 Department of Women’s and Children’s Health, Uppsala University, 751 85 Uppsala, Sweden; 2 Department of Medical Sciences, Uppsala University, 751 85 Uppsala, Sweden

**Keywords:** Biomarkers, Obesity, Intrauterine growth

## Abstract

This study aimed to evaluate the association of maternal adiponectin with infant birth size in 1349 pregnant women at Uppsala University Hospital, Sweden. The mean age of the women was 31.0 years, and 40.9% were nulliparous. Maternal early mid-pregnancy adiponectin was measured in microgram/mL. Linear regression models were performed to evaluate the association between adiponectin and infant birth weight. Logistic regression models were used to evaluate adiponectin in relation to the odds of giving birth to an infant large-for-gestational-age (LGA, infant birth weight standard deviation score > 90th percentile). Adjustments were made for early pregnancy BMI and diabetes mellitus. Prior adjustments, adiponectin was inversely associated with infant birth weight (β − 17.1, 95% confidence interval (CI) − 26.8 to − 7.4 g, *P* < 0.001), and one microgram/mL increase in adiponectin was associated with a 9% decrease in the odds of giving birth to an LGA infant (odds ratio 0.91, CI 0.85–0.97, *P* = 0.006). The associations did not withstand in the adjusted models. We found a significant interaction between adiponectin and infant sex on birth size. This interaction was driven by an inverse association between maternal adiponectin and birth size in female infants, whereas no such association was found in males.

## Introduction

The intrauterine milieu has a considerable impact on fetal growth and later on the birth weight of the infant^1^. Excessive fetal growth is observed among women with obesity^2^ and diabetes mellitus^3^. Increased nutrient availability and elevated insulin levels promoting fetal growth are proposed as mechanisms behind these observations^4^.

A high birth weight is associated with infant morbidity and mortality, and prediction of a high birth weight is therefore of clinical importance. High birth weight infants (≥ 4000 g or ≥ 4500 g)^1^ run an increased risk of complications at delivery, such as asphyxia, birth trauma, meconium aspiration, and stillbirth^5,6^. In the long term, infants born with a high birth weight are at increased risk of developing obesity and diabetes mellitus later in life^7^.

The average birth weight differs between female and male infants, the latter often being heavier^8^. Sex-specific differences in placental function has been suggested as one of the underlying mechanisms behind this fetal growth disparity^1^. For example, the fetal-placental unit of males is proposed to prioritize growth pathways, whereas that of females instead invests in placental reserve capacity^9^. In addition, sex-specific differences in early pregnancy placental biomarkers have been reported, indicating a possible impact of fetal sex on early placentation^10^.

Multiple endocrine and metabolic adaptions take place during pregnancy to secure sufficient energy and nutrient supply to the fetus^11–13^. The first two trimesters of pregnancy are anabolic. During this period, maternal fat stores expand due to increased calorie intake and enhanced lipogenesis^11^. Contrarily, late pregnancy is a catabolic state with decreased insulin sensitivity and hypertriglyceridemia^13^.

The fat tissue is as an important endocrine organ, regulating appetite, energy expenditure, and metabolism through adipokine secretion^14^. The adipokine adiponectin has anti-inflammatory and insulin sensitizing properties^15^. There is an inverse relation between adiponectin levels and BMI; circulating adiponectin levels are lower in obese individuals compared with those with a normal weight^16^. Low adiponectin levels are also associated with diabetes mellitus type 2^17^, and low adiponectin levels early in pregnancy are associated with development of gestational diabetes mellitus^18^. A meta-analysis suggests that adiponectin could be used in risk evaluation of gestational diabetes^19^. In fact, adiponectin and its receptors have been proposed as promising targets for intervention in obese pregnant women with low circulating adiponectin^20^.

First trimester adiponectin levels do not differ from those in non-pregnant individuals. However, adiponectin levels increase during pregnancy. The levels are highest around mid-pregnancy and decrease thereafter^21^. The decrease in adiponectin is seen in both normal weight and obese pregnant women, but is more profound among normal weight individuals^22^.

Previously, a correlation between maternal adiponectin levels and infant birth size has been demonstrated^23–25^. Ahlsson et al.^23^ reports an inverse relation between late-pregnancy adiponectin and maternal fat mass, insulin resistance, and estimated fetal weight in twenty non-diabetic pregnant women. The study did not include maternal BMI in the analyses. Retnakaran et al.^24^ demonstrates a negative association between late-pregnancy adiponectin and birth weight in 472 pregnant women after adjustments for maternal BMI. Likewise, Lowe et al.^25^ reports an inverse association of adiponectin measured in late pregnancy with birth weight in a cohort of 1477 women, with BMI included in the models. These findings suggest that low adiponectin levels might be of importance in the mechanisms linking obesity to excessive fetal growth.

We hypothesized that maternal adiponectin is associated with infant birth size and the risk of giving birth to a large-for-gestational-age (LGA) infant, and that there might be sex-specific differences. This study aimed to investigate the association between circulating levels of adiponectin measured in early mid-pregnancy and birth weight as well as the prevalence of LGA infants in a cohort of 1349 women and child-dyads.

## Material and methods

### Study population

This study was based on data derived from the Uppsala Biobank for Pregnant Women. From 2007, blood samples were collected in this population-based biobank in conjunction with the second-trimester anomaly scan at 16‒20 weeks’ gestation at Uppsala University Hospital, Uppsala, Sweden. Pregnant women who were ≥ 18 years, Swedish-speaking, and without blood-borne disease (HIV, hepatitis C and hepatitis B) were invited to donate a blood sample to the Biobank. The Biobank is a convenience sample since invitations were performed when a research nurse was available. Approximately 30% of the respondents declined to participate. After acceptance to participate, a written informed consent was obtained. Overall, the Biobank is estimated to cover approximately half of Uppsala County´s pregnant population and a sub-population was selected for this study^26^.

We identified all women who had donated a blood sample to the biobank who had body mass index (BMI) ≥ 30.0 kg/m^2^ at the first antenatal visit and a singleton pregnancy (n = 755). Out of these, all women with BMI ≥ 35.0 kg/m^2^ (n = 256) and a random sample of women with BMI 30.0‒34.9 kg/m^2^ (n = 339) were included in the study (n = 595). We also included a random sample of women from the biobank with BMI 25.0‒29.9 kg/m^2^ (n = 437) and BMI 18.5‒24.9 kg/m^2^ (n = 469). Hence, the study population consisted of 1501 pregnant women. Subsequently, we excluded women who gave birth preterm (< 37 weeks + 0 days) (n = 40), post-term (> 41 weeks + 6 days) (n = 107), and cases with missing birth weight (n = 5). Hence, the final study population consisted of 1349 pregnant women who gave birth to singleton, term infants between January 2008 and August 2013.

The study was approved by the Regional Ethical Review Board in Uppsala (Dnr: 2014/353). All research was performed in accordance with relevant national and international guidelines.

### Covariates

A questionnaire was filled out by the women in conjunction with the blood sampling including age, height, weight, smoking habits, medication and chronic disorders. Body mass index was calculated as the weight in kilograms divided by the square of the height in meters. Information on infant birth weight, sex, and gestational age at birth was obtained from the standardized antenatal and delivery medical records. We calculated birth weight standard deviation scores (BWSDS) by the use of Swedish reference standards for birth weight with respect to sex and gestational age^27^.

### Exposure/laboratory analyses

The exposure of this study was the maternal adiponectin level at pregnancy week 16‒20. Blood samples were collected with venesection by a research nurse, centrifuged within 2 h, and stored at -70 °C. All laboratory analyses were performed at the Clinical Chemistry Laboratory, Uppsala University Hospital. Adiponectin was analyzed by commercial sandwich ELISA kits from R&D Systems (Minneapolis, MN, USA) with a limit of detection of 2 ng/mL^28^.

### Statistical methods

The statistical analyses were performed by the use of IBM SPSS Statistics version 28. A nominal two-sided P-value < 0.05 was considered to indicate statistical significance. Non-parametric tests were chosen because the adiponectin levels were not normally distributed according to Shapiro-Wilks test. Differences in adiponectin levels (microgram/mL) between the World Health Organization BMI classes were determined by Kruskal–Wallis test followed by post-hoc paired tests with Bonferroni correction for multiple testing. Spearman’s correlation coefficients were calculated to evaluate the relations between early pregnancy BMI, maternal age, adiponectin, and infant birth size*.* Multiple linear regression models were used to evaluate the association of adiponectin levels with infant birth weight in grams and BWSDS. The observed standardized residuals of the linear regression models were normally distributed. The VIF (variance inflation factor) values were ≤ 1.4, indicating that multicollinearity was not an issue in the model. Logistic regression models were performed to evaluate adiponectin levels in relation to the odds of giving birth to an LGA infant (BWSDS > 90^th^ percentile). Covariates included in the models evaluating birth size were early pregnancy BMI and pregestational or gestational diabetes mellitus (yes or no). These covariates were identified by drawing and analyzing a directed acyclic graph (DAG) (Supplementary Fig. S1 online)^29^. Variables were included in the DAG if they were known to be associated with the exposure and outcome, or if they were considered clinically relevant. We also performed sensitivity analyses including only women without pregestational or gestational diabetes mellitus. In the sensitivity analyses, adjustment were made for early pregnancy BMI. To investigate any sex-specific differences, we performed an interaction analysis on the interaction between adiponectin and infant sex on birth weight. The interaction analysis was adjusted for early pregnancy BMI and diabetes mellitus (pregestational or gestational). This interaction analysis was followed by linear and logistic regression analyses evaluating the relation between adiponectin and birth size on female and male infants separately.

## Results

### Study population

The study population consisted of 1349 mother and child-dyads. The women were between 18 and 48 years of age, 552 (40.9%) were pregnant with their first child, and 915 (67.8%) had either overweight (BMI ≥ 25) or obesity (BMI ≥ 30). The study population characteristics are presented in Table 1.

**Table 1:** Study population characteristics.

	n	1349
Women	Age, years (mean, min‒max)	31.0 (18‒48)
	Country of birth within EU, n (%)	1235 (91.5)
	BMI at first antenatal visit, kg/m^2^ (mean ± SD)	28.5 ± 6.0
	Nulliparous, n (%)	552 (40.9)
	Smoking at first antenatal visit, n (%) ^a^	89 (6.8)
	Asthma, n (%)	132 (9.8)
	Inflammatory bowel disease, n (%)	10 (0.7)
	Hypertension, n (%)	9 (0.7)
	Hypothyroidism, n (%)	37 (2.7)
	Diabetes mellitus, n (%)	2 (0.1)
	Epilepsy, n (%)	13 (1.0)
	Rheumatic disease, n (%)	2 (0.1)
	Renal disease, n (%)	5 (0.4)
	Gestational diabetes, n (%)	24 (1.8)
	Gestational hypertension, n (%)	56 (4.2)
	Preeclampsia, n (%)	27 (2.0)
Infants	Female, n (%)	650 (48.2)
	Birth weight, g (mean ± SD)	3684 ± 494
	LGA, n (%)	168 (12.5)
	SGA, n (%)	51 (3.8)
	Gestational age at birth, days (mean ± SD)	279 ± 8

*BMI* body mass index, *LGA* large-for-gestational-age (birthweight standard deviation score (BWSDS) above the 90th percentile), *SGA* small-for-gestational-age (BWSDS below the 10th percentile).

^a^Data missing in 47 individuals.

### Early mid-pregnancy adiponectin in relation to maternal characteristics

The overall range of adiponectin was 0.52‒18.20 µg/mL. The adiponectin measures according to BMI classes are presented in Supplementary Table S1. There was a decrease in circulating adiponectin with increasing BMI, although the difference between BMI classes was not always statistically significant (Supplementary Table S1 online). Early pregnancy adiponectin levels correlated negatively with BMI (r = − 0.62) (Supplementary Table S2 online). There was no correlation between adiponectin and maternal age (Supplementary Table S2 online).

### Early mid-pregnancy adiponectin in relation to infant birth weight

For every microgram/mL increase in adiponectin there was an associated decrease in birth weight of 17.1 g (95% confidence interval (CI) − 26.8 to − 7.4 g, *P* < 0.001) (Table 2). However, no association was seen in the model adjusted for early pregnancy BMI and diabetes mellitus (pregestational or gestational) (β 0.3, CI − 11.0 to 11.7, *P* = 0.953) (Table 2). The results were similar in the analysis evaluating BWSDS (Table 2).

**Table 2:** Associations between maternal adiponectin levels and infant birth size.

Outcome	Unadjusted model			Adjusted model^a^		
	β	CI	*P*	β	CI	*P*
Birth weight (g)	− 17.1	− 26.8 to − 7.4	< 0.001	0.3	− 11.0 to 11.7	0.953
Birth weight standard deviation score (BWSDS)	− 0.03	− 0.05 to − 0.02	< 0.001	0.00	− 0.02 to 0.02	0.945

Data are B coefficients (β) and (95% confidence interval (CI)) for the change in outcome per unit increase in adiponectin (microgram/mL).

Data were analyzed using linear regression models. Separate regression analyses were performed for each outcome.

^a^Adjustments were made for early pregnancy BMI and diabetes mellitus (pregestational or gestational).

An increase of adiponectin by one microgram/mL was associated with a 9% decrease in the odds of having an LGA infant (OR 0.91, CI 0.85–0.97, *P* = 0.006), but the association did not withstand in the adjusted model (OR 1.00, CI 0.93–1.08, *P* = 0.976) (Table 3).

**Table 3:** Associations between maternal adiponectin levels and the likelihood of giving birth to an infant large-for-gestational-age (LGA).

Outcome	Unadjusted model			Adjusted model^a^		
	OR	CI	*P*	OR	CI	*P*
**LGA**	0.91	0.85–0.97	0.006	1.00	0.93–1.08	0.976

Data are odds ratios (OR) (95% confidence interval (CI)) for the change in outcome per unit increase in adiponectin (microgram/mL).

Data were analyzed using logistic regression models.

^a^Adjustments were made for early pregnancy BMI and diabetes mellitus (pregestational or gestational).

In the sensitivity analyses including only women without diabetes mellitus, the results were similar (Supplementary Tables S3 and S4 online).

### Early mid-pregnancy adiponectin in relation to birth weight in female and male infants

An interaction between adiponectin and infant sex on birth weight was demonstrated; the effect on birth weight of one microgram/mL increase in adiponectin was 20.2 g lower for females versus males (CI − 39.4 to − 1.0, *P* = 0.039) (Table 4).

**Table 4:** Interaction between maternal adiponectin levels (microgram/mL) and infant sex on birth weight in grams.

	β	CI	*P*
Main effects			
Adiponectin	9.3	− 4.9 to 23.4	0.199
Infant sex (female)	− 0.8	− 104.7 to 103.0	0.987
Interaction term			
Adiponectin x infant sex	− 20.2	− 39.4 to − 1.0	0.039

Data are B coefficients (β) and (95% confidence interval (CI)) for the change in outcome per unit increase in adiponectin (microgram/mL).

Data were analyzed using linear regression models.

Adjustments were made for early pregnancy BMI and diabetes mellitus (pregestational or gestational).

Stratified analyses in each sex revealed an inverse association between adiponectin and birth size prior to adjustments in female infants (Supplementary Tables S5 and S6 online). One microgram/mL increase in adiponectin was associated with a decrease in birth weight of 29.2 g (CI − 43.6 to − 14.8 g, *P* < 0.001) in female infants (Supplementary Table S5 online). The association did not withstand after adjustments. An increase of adiponectin by one microgram/mL was also associated with a 12% decrease in the odds of having an LGA infant (OR 0.88, CI 0.80–0.97,* P* = 0.013) among female infants (Supplementary Table S6 online). In male infants, there was no association between adiponectin and birth size (Supplementary Tables S5 and S6 online).

## Discussion

The results of this study demonstrated an inverse relation between early mid-pregnancy adiponectin levels and infant birth weight and the likelihood of giving birth to an LGA infant. However, adiponectin levels were not associated with birth weight or LGA when early pregnancy BMI was included in the models. The results from the sex-stratified analyses suggested that low circulating adiponectin might be a link between maternal adiposity and increased birth size in females, but not in males.

The sensitivity analysis including only non-diabetic pregnant women showed similar results indicating that insulin resistance (above the cut-off value for diabetes diagnosis) is probably not the main mechanism linking low circulating adiponectin to excessive fetal growth and subsequently increased birth weight.

We found an inverse association between adiponectin and birth size in female infants, but not in males. To the best of our knowledge, this is the first study to evaluate the relation between maternal adiponectin levels and infant birth size in females and males separately. We have previously reported on sex-specific differences regarding maternal blood-based proteins measured in early mid-pregnancy in relation to infant birth size^30^. Hence, it seems that sex-specific adaptions to in-utero environmental changes occur, but the precise mechanisms behind are unknown. Nonetheless, altered placental function and feto-placental hormonal changes may contribute to the sex-specific divergences in adaption to intra-uterine milieu variations^9^.

An inverse association of maternal adiponectin with infant birth size has been described by other investigators^31,32^. Migda et al.^31^ reports lower first trimester adiponectin levels among women delivering macrosomic (> 4000 g) and LGA-infants compared with controls. The study did not adjust for maternal BMI. Wang et al.^32^ measured maternal adiponectin right before delivery in 100 mother-and-child dyads. The results demonstrate decreased adiponectin levels in women giving birth to macrosomic infants compared with controls. Of note, BMI was not included in the analyses. Moreover, the authors investigated placental adiponectin expression and report reduced adiponectin expression in placentas from women delivering macrosomic infants compared with control mothers. In line with these findings, early pregnancy adiponectin has been suggested as a feasible biomarker in prediction of macrosomic infants^33^. Interestingly, a study investigating adiponectin measures multiple times during pregnancy reports that women giving birth to LGA infants had the largest decrease in adiponectin throughout their pregnancy^34^.

The mechanisms underpinning the association between low circulating maternal adiponectin and increased birth weight are not fully understood. However, several explanations have been proposed on this matter. Fetal growth, and subsequently infant birth weight, is dependent on adequate nutrient transport from the placenta. Maternal adiponectin does not cross the placenta to the fetal circulation, but both adiponectin^35^ and the adiponectin receptors ADIPOR1 and ADIPOR2 are expressed by placental cells^36^. Actually, expression of ADIPOR2 in the placenta differs between obese and non-obese women, with a down-regulation among obese pregnant individuals^37^. By these receptors, adiponectin could influence placental nutrient transport to the fetus and thereby regulate fetal growth. It is shown that adiponectin inhibits the expression of glucose and amino acid transporters in placental cells, which could limit nutrient availability for the fetus. In addition, adiponectin can induce placental apoptosis. Hence, placental function, and subsequently fetal growth, could be affected by maternal adiponectin through inhibition of nutrient transport expression as well as apoptosis activation^38^.

The relation between adiponectin and birth size has been investigated in animal studies. Pregnant mice were given adiponectin infusions and fetal growth was evaluated. The results show an 18% decrease in fetal weights among mice given adiponectin compared with controls^39^. Another study comparing adiponectin infusion with sham injection in obese mice demonstrates reduced fetal size in mice administered adiponectin compared with controls, strengthening the hypothesis that adiponectin could be a link between maternal fat tissue and fetal growth^40^.

Our results showed an inverse relation between adiponectin and maternal BMI, which is in line with previous findings^22,25,41,42^. In addition, we found that maternal BMI had a stronger correlation with birth size than did adiponectin.

This study has limitations and strengths. The cross-sectional design of this study is a limitation since we were not able to investigate a possible longitudinal association between adiponectin and birth weight. Previous studies investigating the relation between maternal adiponectin levels and infant birth size are performed on smaller study samples compared with this present study, especially those evaluating early pregnancy adiponectin. This study contributes to the field by confirming the association between maternal adiponectin measured in early pregnancy and birth size in a large population-based cohort of pregnant women. It should be noted that there was no independent association between adiponectin and birth weight, the association disappeared when maternal BMI was added to the models. To the best of our knowledge, this is the largest study evaluating maternal early pregnancy adiponectin levels in relation to infant birth size and the likelihood of giving birth to an LGA infant, including sex-stratified analyses. Yet another strength is the population based setting, increasing the generalizability of the results.

## Conclusion

This study confirmed previous work showing an inverse relation between maternal early mid-pregnancy adiponectin and infant birth weight and the likelihood of giving birth to an LGA infant. However, the association between adiponectin and birth size was not independent of maternal BMI. There were also sex-specific differences, the association between adiponectin and birth size was observed only in female infants, not in males. Hence, adiponectin could possibly be a factor linking maternal fat accumulation mechanistically to fetal growth and subsequently birth size in female infants.

### Supplementary Information

Supplementary Information.

## Data Availability

The datasets generated during and/or analyzed during the current study are available from the corresponding author on reasonable request.
