# Supplementary material for: Maternal early mid-pregnancy adiponectin in relation to infant birth weight and the likelihood of being born large-for-gestational-age
Source: Sci Rep. 2023 Nov 27;13:20919. doi: 10.1038/s41598-023-48027-2 (PMC10684871; doi:10.1038/s41598-023-48027-2)
Supplement: Supplementary file 1 — Supplementary Information. [file 41598_2023_48027_MOESM1_ESM.pdf]

Supplementary information file

Maternal early mid-pregnancy adiponectin in relation to infant birth weight and the likelihood of being born large-for-gestational-age

Emelie Lindberger MD PhD[1*], Anders Larsson MD, PhD[2], Theodora Kunovac Kallak PhD[1], Inger Sundström Poromaa MD PhD[1], Anna-Karin Wikström MD PhD[1], Anna Österroos MD[1], Fredrik Ahlsson MD PhD[1]

[1] Department of Women’s and Children’s Health, Uppsala University, 751 85 Uppsala, Sweden

[2] Department of Medical Sciences, Uppsala University, 751 85 Uppsala, Sweden

Supplementary Figure S1. Directed acyclic graph (DAG) used for selection of covariates.

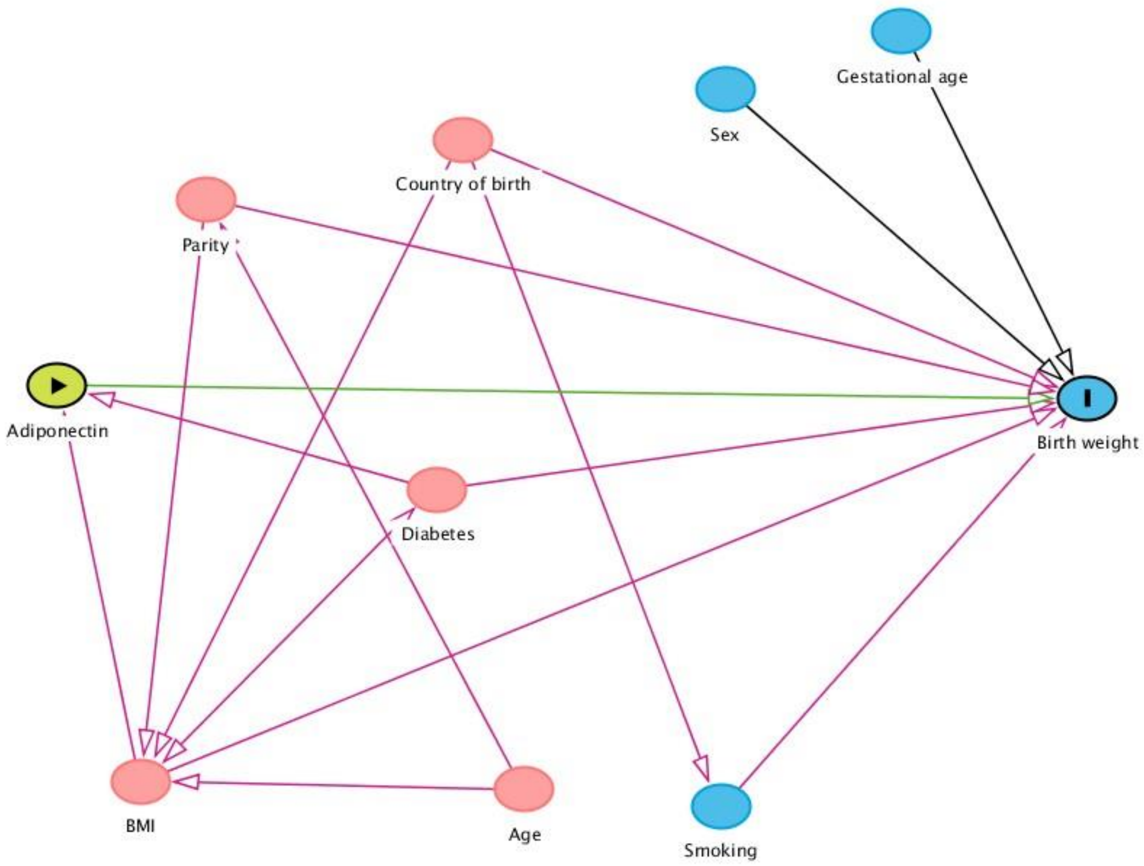

Supplementary Table S1. Adiponectin measures in relation to BMI classes defined by the WHO.

		Adiponectin (microgram/mL)			
**BMI, WHO classification (kg/m^2)**	**N (%)**	**Min**	**Max**	**Mean**	**SD**
<18.5 (underweight)	0 (%)	-	-	-	-
18.5–24.9 (normal weight)	434 (32.2)	1.4	18.0	6.1[a]	2.3
25.0–29.9 (overweight)	404 (29.9)	0.5	18.2	5.7[a]	2.9
30.0–34.9 (obesity class I)	290 (21.5)	0.7	8.6	2.8[b]	1.2
35.0–39.9 (obesity class II)	162 (12.0)	0.7	8.2	2.7[c]	1.3
≥40.0 (obesity class III)	59 (4.4)	1.0	4.3	2.3[d]	1.0

[a] significantly different from all other WHO BMI-classes, *P* =0.001. Kruskal-Wallis test followed by post-hoc paired tests with Bonferroni correction.

[b] significantly different from all other WHO BMI-classes, *P* <0.001, except obesity class II and III. Kruskal-Wallis test followed by post-hoc paired tests with Bonferroni correction.

[c] significantly different from all other WHO BMI-classes, *P* <0.001, except obesity class I and III. Kruskal-Wallis test followed by post-hoc paired tests with Bonferroni correction.

[d] significantly different from all other WHO BMI-classes, *P* <0.001, except obesity class I and II. Kruskal-Wallis test followed by post-hoc paired tests with Bonferroni correction.

BMI, body mass index kg/m^2; SD, standard deviation

Supplementary Table S2. Spearman correlation coefficients for the associations between characteristics of the women and infant birth size.

	**Age**	**Adiponectin**	**Infant BWSDS**	**Birth weight**
**Early pregnancy BMI**	-0.01	-0.62*	0.16*	0.16*
**Age**		0.04	0.02	0.02
**Adiponectin**			-0.11*	-0.11*
**Infant BWSDS**				1.00*

BWSDS, birth weight standard deviation score; BMI, body mass index.

*P <0.01.

Supplementary Table S3. Associations between maternal adiponectin levels and infant birth size among women without diabetes mellitus (n=1323).

**Outcome**	**Unadjusted model**			**Adjusted model** [a]		
	β	CI	*P*	β	CI	*P*
**Birth weight (g)**	-14.9	-24.6 to -5.2	0.003	0.2	-11.2 to 11.6	0.970
**Birth weight standard deviation score**	-0.03	-0.05 to -0.01	0.002	0.00	-0.02 to 0.02	0.974

Data are B coefficients (β) and (95% confidence interval (CI)) for the change in outcome per unit increase in adiponectin (microgram/mL).

Data were analyzed using linear regression models.

[a] Adjustments were made for early pregnancy BMI.

Supplementary Table S4. Associations between maternal adiponectin levels and the likelihood of giving birth to an infant large-for-gestational-age (LGA) among women without diabetes mellitus (n=1323).

**Outcome**	**Unadjusted model**			**Adjusted model** [a]		
	OR	CI	*P*	OR	CI	*P*
**LGA**	0.93	0.87 – 0.99	0.028	1.00	0.93 − 1.08	0.938

Data are odds ratios (OR) (95% confidence interval (CI)) for the change in outcome per unit increase in adiponectin (microgram/mL).

Data were analyzed using logistic regression models.

[a] Adjustments were made for early pregnancy BMI.

Supplementary Table S5. Associations between maternal adiponectin levels and infant birth weight, female and male infants analyzed separately.

**Females (n =650)**	**Unadjusted model**			**Adjusted model** [a]		
	β	CI	*P*	β	CI	*P*
**Birth weight (g)**	-29.2	-43.6 to -14.8	<0.001	-3.6	-20.1 to 12.9	0.669
**Birth weight standard deviation score**	-0.06	-0.08 to -0.03	<0.001	-0.01	-0.04 to 0.02	0.661

**Males (n =699)**	**Unadjusted model**			**Adjusted model** [a]		
	β	CI	*P*	β	CI	*P*
**Birth weight (g)**	-8.0	-21.1 to 5.0	0.228	1.9	-13.6 to 17.5	0.808
**Birth weight standard deviation score**	-0.01	-0.04 to 0.01	0.232	0.00	-0.02 to 0.03	0.795

Data are B coefficients (β) and (95% confidence interval (CI)) for the change in outcome per unit increase in adiponectin (microgram/mL).

Data were analyzed using linear regression models.

[a] Adjustments were made for early pregnancy BMI and diabetes mellitus (pregestational or gestational).

Supplementary Table S6. Associations between maternal adiponectin levels and the likelihood of giving birth to an infant large-for-gestational-age (LGA), female and male infants analyzed separately.

**Females (n =650)**	**Unadjusted model**			**Adjusted model** [a]		
	OR	CI	*P*	OR	CI	*P*
**LGA**	0.88	0.80 – 0.97	0.013	1.02	0.91 – 1.13	0.740

**Males (n =699)**	**Unadjusted model**			**Adjusted model** [a]		
	OR	CI	*P*	OR	CI	*P*
**LGA**	0.94	0.86 – 1.03	0.164	0.98	0.88 – 1.09	0.653

Data are odds ratios (OR) (95% confidence interval (CI)) for the change in outcome per unit increase in adiponectin (microgram/mL).

Data were analyzed using logistic regression models.

[a] Adjustments were made for early pregnancy BMI and diabetes mellitus (pregestational or gestational).
